# Fracture Closure Empirical Model and Theoretical Damage Model of Rock under Compression

**DOI:** 10.3390/ma16020589

**Published:** 2023-01-07

**Authors:** Yifan Chen, Hang Lin, Shijie Xie, Rihong Cao, Shuwei Sun, Wenhua Zha, Yixian Wang, Yanlin Zhao, Huihua Hu

**Affiliations:** 1 School of Resources and Safety Engineering, Central South University, Changsha 410083, China; 2 School of Energy and Mining Engineering, China University of Mining and Technology (Beijing), Beijing 100083, China; 3 School of Civil and Architectural Engineering, East China University of Technology, Nanchang 330013, China; 4 School of Civil Engineering, Hefei University of Technology, Hefei 230009, China; 5 School of Energy and Safety Engineering, Hunan University of Science and Technology, Xiangtan 411201, China

**Keywords:** rock damage, deformation evolution, fracture closure, constitutive model, uniaxial compression test, triaxial compression test

## Abstract

The rock or rock mass in engineering often contains joints, fractures, voids, and other defects, which are the root cause of local or overall failure. In response to most of the current constitutive models that fail to simulate the nonlinear fracture compaction deformation in the whole process of rock failure, especially brittle rocks, a piecewise constitutive model was proposed to represent the global constitutive relation of rocks in this study, which was composed of the fracture compaction empirical model and the damage statistical constitutive model. The fracture empirical compaction model was determined by fitting the expressions of fracture closure curves of various rocks, while the rock damage evolution equation was derived underpinned by the fracture growth. According to the effective stress concept and strain equivalence hypothesis, the rock damage constitutive model was deduced. The model parameters of the fracture compaction empirical model and damage statistical constitutive model were all calculated by the geometrical characteristics of the global axial stress–strain curve to guarantee that the models are continuous and smooth at the curve intersection, which is also simple and ready to program. Finally, the uniaxial compression test data and the triaxial compression test data of different rocks in previous studies were employed to validate the models, and the determination coefficient was used to measure the accuracy. The results showed great consistency between the model curves and test data, especially in the pre-peak stage.

## 1. Introduction

**Figure 1:**
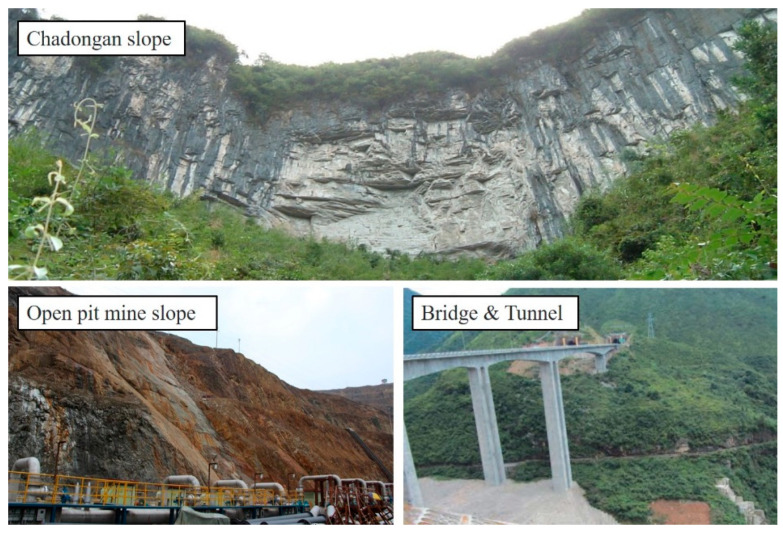
Common rock engineering.

**Figure 2:**
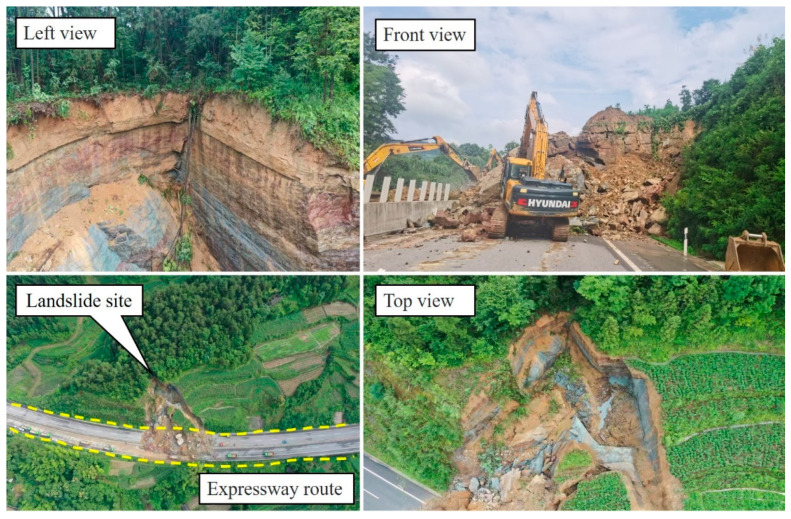
The highway geological landslide.

Rock is a material ubiquitous in mines, expressways, tunnels, dams, slopes, and other rock mass engineering [1,2,3], as shown in Figure 1. The construction and operation of engineering are closely related to the physical and mechanical properties of rocks [4,5,6], whereas rock is essentially a kind of heterogeneous medium, which is a complex mineral aggregate formed under physical and chemical actions after a long geological process [7,8]. It is mainly composed of a variety of mineral grains and cements and contains a large number of defects, such as joints, micro-cracks, pores, holes, and faults [9,10,11]. They are also regarded as the root of macroscopic fractures, which may cause severe rock mass instability under the actions of natural weathering or engineering disturbance, or even evolve into geological disasters such as landslides (see Figure 2), and it will have a great impact on the environment [12,13,14].

It is this complexity that makes the relationship between the stress and strain of rock one of the most concerned problems in geotechnical engineering [15,16,17]. In 1948, Cook [18] put forward the concept of the whole stress–strain process of rock, and divided the actual typical uniaxial compression test curve of rock into the compaction stage, the elastic stage, the plastic hardening stage, and the strain softening stage, which simulate the whole deformation process and have become powerful tools to study the failure mode and mechanism of rocks [19,20]. Since then, global scholars and engineers began to study the other rock constitutive models in addition to the classical elastic–plastic model [21,22].

The establishment of a rock damage constitutive model based on the randomly distributed internal defects is another effective approach to study the constitutive relation of rock materials [23]. Experimental and numerical evidence shows that rock failure is a gradual process [24], which is the result of the propagation of random distributed primary fractures and the generation and development of new fractures [25,26]. Such randomness can be indicated by statistical methods. Krajcinovic and Silva [27] combined the continuous damage theory and statistical strength theory, which has inspired a new approach for the research of rock constitutive models. The statistical damage constitutive model for rocks with strain softening behavior was also put forward based on the maximum entropy distribution [28,29]. In addition, Li and Liao [30] assumed that the strengths of elements obey the unified strength theory and proposed a statistical damage constitutive model under the complex stress state. Liu et al. [31] established a new damage constitutive model for the rock mass with non-persistent joints. Xu et al. [32,33] established the thermal-mechanical coupling damage constitutive model of rock by the Weibull distribution. Beyond that, Xu et al. [34] also used the damage variable correction factor, $\delta =\sqrt{\sigma_{r}/\sigma_{c}}$, to establish the damage statistical constitutive model reflecting the residual strength. Feng et al. [35] established the strain-softening damage model of the rocks with defect growth based on damage evolution. Chen et al. [36] built a new statistical damage constitutive model underpinned by the Hoek–Brown strength criterion and damage theory. Based on the disturbed state concept (DSC) for the shear deformation of rock joints, Xie et al. [37] deduced the DSC shear constitutive model to connect the mechanical behavior of micro-units with the macroscopic joint shear deformation characteristics [38]. Meanwhile, they also proposed a novel constitutive model to predict the shear deformation behavior of the discontinuity from the perspective of landslide prevention [39]. In fact, rock volume constantly changes with particle breakage and pore extrusion when subjected to initial loads, causing nonlinearity at compaction [40,41]. However, the existing damage statistical constitutive models have rarely considered the nonlinearity caused by fracture closure and are initiated directly with linear deformation, which fail to accurately reflect the pre-peak deformation [42,43].

To address the issue, a piecewise constitutive model was proposed for the representation of the whole axial stress–strain relation of rock under compression, which is divided into the compaction model and the damage model by the fracture closure point. First, the stress increment tendency with the strain during the fracture closure stage was analyzed, which was fitted by a power function. The compaction model was gained by solving function parameters according to the initial modulus and elasticity modulus. Then, the damage variable of the rock material was defined as the ratio of the rock damaged component to the rock entirety, and the damage evolution equation was derived from the fracture growth pattern, which was subsequently applied to infer the damage constitutive model on the basis of the effective stress concept and strain equivalence hypothesis. Ultimately, the piecewise constitutive model considering the deformation feature during fracture closure was achieved, which was fitted from the existing uniaxial and triaxial compression test results. Compared with the model by Feng et al. [35], this newly proposed model shows a more concise expression in the compaction stage and residual stage, and the derivation is easier to understand. It also provides a certain reference for the embedment of the rock constitutive model under compression conditions in numerical software.

## 2. Fracture Development Stages and Progressive Failure of Rock

**Figure 3:**
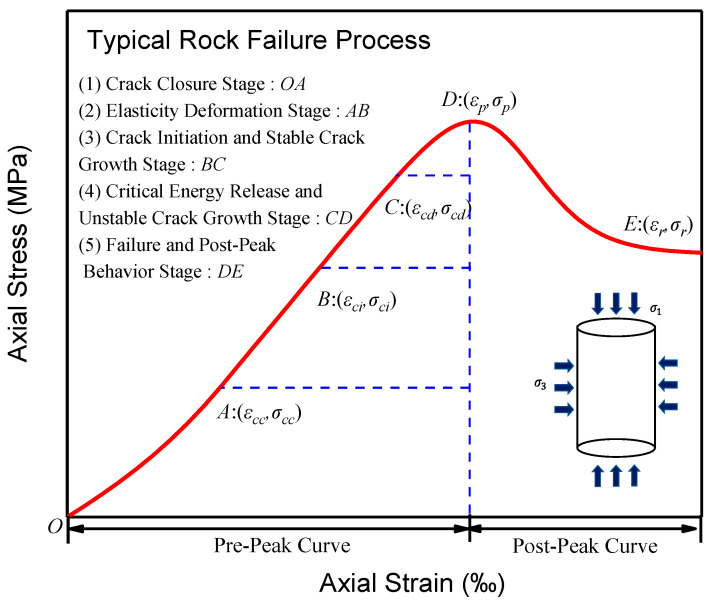
The typical rock failure process.

From a general view on the typical rock failure process, a complete axial stress–strain curve includes the pre-peak curve and the post-peak curve [44,45], as shown in Figure 3. The pre-peak curve can further be divided into four stages by fracture development, simultaneously gaining four stress thresholds [46,47,48]: fracture closure stress *σ_cc_*, fracture initiation stress *σ_ci_*, rock damage stress *σ_cd_*, and peak stress *σ_p_*. The post-peak curve starts at the peak stress *σ_p_* and ends at the residual stress *σ_r_*. The pre-peak curve can further be divided into five stages:

1. Fracture closure stage. In the literal sense, fractures close during the initial loading process, and the stress–strain response is nonlinear, the regional extent of which is dependent on the initial fracture density and geometrical characteristics of the fracture population [49].
2. Elasticity deformation stage. Rock can be regarded as a dense material in this stage, and the mechanical behavior is linear-elastic, reflected by a straight line in the stress–strain curve. The elastic mechanics constants of rock such as elastic modulus and Poisson’s ratio are usually determined by this line.
3. Fracture initiation and stable fracture growth stage. New fractures initiate and propagate at a steady rate.
4. Critical energy release and unstable fracture growth stage. Dilatancy occurs to the rock. The fractures grow precariously, and the old fractures and the new fractures interweave, causing macroscopical failure of rock.
5. Failure and post-peak behavior stage. Rock stress drops rapidly but retains a certain bearing capacity provided by friction.

In view of the limitation that the current statistical damage constitutive model cannot reflect well the mechanical properties of pre-peak deformation of rocks, especially brittle rocks such as coal, this paper constructed a novel rock constitutive model consisting of three parts: fracture closure model, fracture growth model, and residual strength model, and the detailed steps are described in the following content.

## 3. Establishment Process of Piecewise Constitutive Model of Rock

### 3.1. Fitting of Empirical Model in Fracture Closure Stage

**Figure 4:**
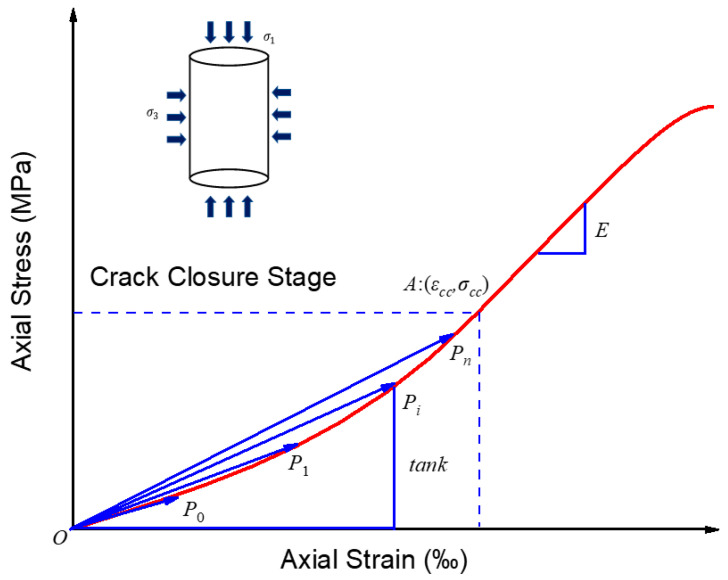
The deformation characteristics of fracture closure curve.

As previously described, natural rocks are essentially composed of various mineral particles, while the complexity of the cementing mode and the randomness of cementing position are conducive to the presence of pores and defects inside the rocks. Under the compressing stress, these pores and defects are prone to deformation and closure, which causes the axial stress–strain curve to show a downward convex characteristic at the inception of loading, as shown in Figure 4. According to the rock damage evolution characteristics, the rock damage degree is so small that it can be ignored in the fracture closure stage [50,51]. Thus, the empirical model of rock fracture closure was directly achieved by the mathematical method.

**Figure 5:**
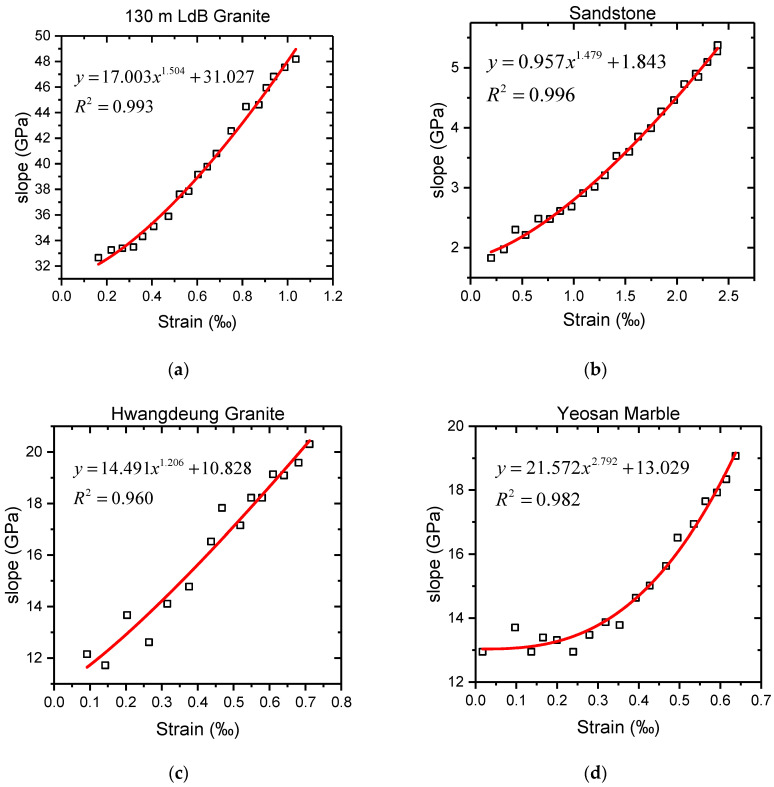
The variation law of *k*. (**a**) Eberhardt et al. [49]; (**b**) Gao et al. [52]; (**c**) Chang et al. [53]; (**d**) Chang et al. [53].

Under the assumption that the rock axial stress–strain curve is continuous and differentiable, if a point *P_i_* (*ε_i_*, *σ_i_*) is taken on the curve *OA* and point *P_i_* is connected to origin *O*, the slope of *OP_i_* can be calculated by the coordinates of point *P_i_*, *k* = *σ_i_*/*ε_i_*, which shows an increasing trend with the increase in axial strain. Based on the triaxial test data of various rock samples in previous studies [49,52,53], the variation law of *k* is presented in Figure 5. According to the variation characteristics, the change in the value of *k* from the fracture closure stage to the elasticity deformation stage is a gradually increasing process and the increment is also enlarging, which can be fitted by a power function as shown in Equation (1). All the values of *R*^2^ in Figure 5 are greater than 0.95, demonstrating the effectiveness of this fitting. Therefore, the change in the value of *k* with strain *ε* can be expressed by Equations (1) and (2) and can be obtained by simple formula manipulation:

(1) $$k=\frac{\sigma_{1}}{\varepsilon_{1}}=a\varepsilon_{1}^{b}+c$$

(2) $$\sigma_{1}=a\varepsilon_{1}^{b+1}+c\varepsilon_{1}$$

where *a*, *b*, and *c* are parameters to be solved.

The derivative of the axial stress–strain curve at *ε*_1_ = 0 is namely the initial modulus *E_ini_*, and Equation (2) can be changed into the following:

(3) $$\begin{array}{cc} \frac{d\sigma_{1}}{d\varepsilon_{1}}=a\left(b+1\right)\varepsilon_{1}+c & \end{array}$$

When *ε*_1_ = 0, the value of parameter *c* can be solved.

(4) $$c=E_{ini}$$

At the point of fracture closure stress *σ_cc_* (*ε*_1_ = *ε_cc_*), the relationship of Equation (5) is obtained, and the derivative of the axial stress–strain curve equals elastic modulus *E*, which means:

(5) $$\sigma_{cc}=a\varepsilon_{cc}^{b+1}+c\varepsilon_{cc}$$

(6) $$a\left(b+1\right)\varepsilon_{cc}+c=E$$

By solving simultaneous equations of Equations (4)–(6), the specific values of parameters *a* and *b* can be determined, as shown in Equations (7) and (8).

(7) $$a=\frac{\sigma_{cc}/\varepsilon_{cc}-E_{ini}}{\varepsilon_{cc}^{b}}$$

(8) $$b=\left(E-\frac{\sigma_{cc}}{\varepsilon_{cc}}\right)/\left(\frac{\sigma_{cc}}{\varepsilon_{cc}}-E_{ini}\right)$$

### 3.2. Derivation of Rock Damage Constitutive Model Underpinned by Fracture Growth

When the rock constitutive model is established from the perspective of damage, the equivalent strain hypothesis is inevitably involved [54]. Since being proposed by Lemaitre in 1985, it has made significant contributions to the rock damage constitutive model establishment. Based on the continuity factor concept proposed by Kachanov [55], the rock material was assumed to consist of a theoretically infinite number of micro units with the same size (i.e., rock micro-units that are mathematically small enough), which can be divided into a damaged component and undamaged component. In this case, the damage variable was first defined as the ratio of the damaged cross-sectional area to the total cross-sectional area [56], and it can be described by Equation (9).

(9) $$D=\frac{A_{dam}}{A_{tol}}=1-\frac{A_{und}}{A_{tol}}$$

where *D* is rock damage variable; *A_dam_* is the damaged area in the total cross-sectional area of the rock; *A_und_* is the undamaged area in the total cross-sectional area of the rock; *A_tol_* is the total cross-sectional area of the rock.

**Figure 6:**
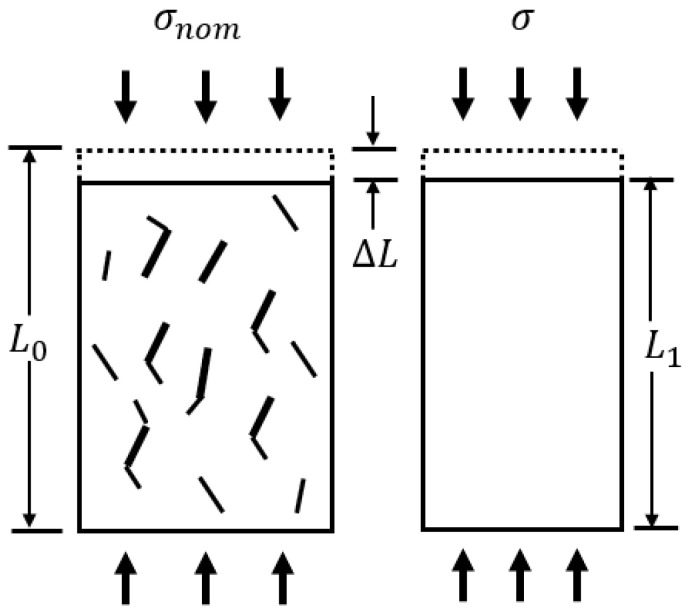
The strain equivalence hypothesis diagram.

Subsequently, Lematire [54] established the strain equivalence hypothesis, which pointed out that the strain caused by nominal stress was equal to that caused by the actual stress (as shown in Figure 6), as presented in Equation (10).

(10) $$\frac{\sigma_{nom}}{E_{dam}}=\frac{\sigma }{E}$$

where *σ_nom_* is the nominal stress applied on the damage rock material, *σ_nom_* = *F*/*A_tol_*; *E_dam_* is the elastic modulus of the damage rock material; *σ* is the actual stress the damaged rock material is subjected to, *σ_act_* = *F*/*A_und_*; *E* is the elastic modulus of the intact rock material; *F* is the applied axial force.

By combining Equations (9) and (10), as well as Hooke’s law, the rock damage model can be expressed as

(11) $$\sigma_{1}=E\varepsilon_{1}\left(1-D\right)$$

**Figure 7:**
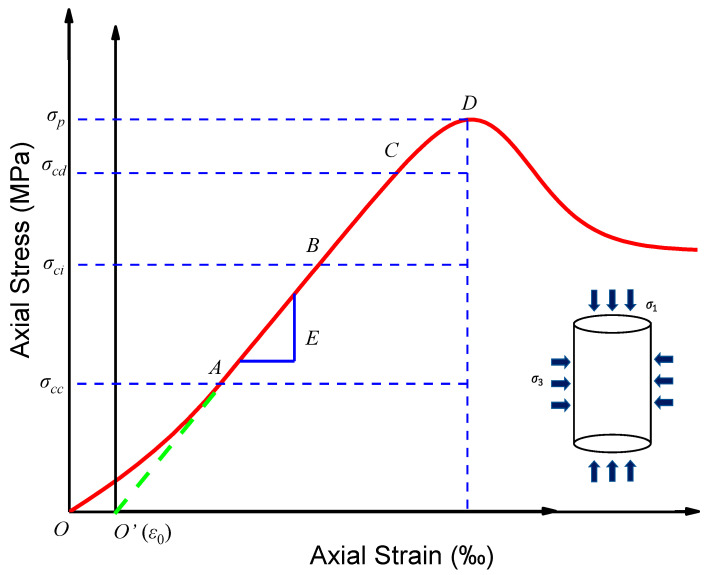
Stress–strain curve for the new coordinate system.

Apparently, the rock damage constitutive models originating from Equation (11) neglect the fracture closure stage and start with the elasticity deformation stage in the theoretical axial stress–strain curve, failing to reflect the nonlinear characteristic fracture closure stage. To address this issue, the linear elastic curve was first reversely prolonged to intersect the horizontal axis at point *O*’ (*ε*_0_,0). If elastic modulus *E* and closure stress *σ_cc_* are known, the value of *ε*_0_ can be determined by Equation (12). Then, by shifting the vertical axis so that the origin *O* locates at point *O*’, a new coordinate system was set-up, as shown in Figure 7. Therefore, Equation (11) in the new coordinate system can be recast as

(12) $$\varepsilon_{0}=\varepsilon_{cc}-\sigma_{cc}/E$$

(13) $$\sigma_{1}=E\left(\varepsilon_{1}-\varepsilon_{0}\right)\left(1-D\right)$$

If the rock material is divided into *n* units of equal area including damaged units and undamaged units only, then the damage variable *D* can be rewritten as Equation (14).

(14) $$D=\frac{n_{dam}}{n}=1-\frac{n_{und}}{n}$$

where *n_dam_* and *n_und_* are the quantity of damaged units and undamaged units, respectively.

According to the progressive failure concept of rock, damage first occurs at the weak point inside the rock, and constantly initiates, propagates, and connects, eventually leading to rock failure. Therefore, the damage evolution process of rock can be regarded as a growth process of damaged units. The quantity and growth rate of damaged units determine the failure state of the rock. The increase in damaged unit quantity can accelerate the growth rate of new damaged units, which will increase the quantity of damaged units in turn. Based on the population growth retardation model in biology, the growth equation of the damaged unit [57] is:

(15) $$\frac{\partial n_{dam}}{\partial \varepsilon }=\upsilon n_{dam}\left(1-\frac{n_{dam}}{n}\right)$$

where *υ* is the intrinsic growth rate of the damaged units.

By substituting Equation (14) into Equation (15) and through integral transformation, the following equation can be gained:

(16) $$D=\frac{1}{1+\exp\left(\lambda -\upsilon \varepsilon \right)}$$

where *λ* is related to the degree of initial damage; *υ* is associated with the rate of damage growth.

By substituting Equation (16) into Equation (13), Equation (17) can be obtained.

(17) $$\sigma_{1}=E\left(\varepsilon_{1}-\varepsilon_{0}\right)\left\{1-\frac{1}{1+\exp\left[\lambda -\upsilon \left(\varepsilon_{1}-\varepsilon_{0}\right)\right]}\right\}$$

As the theoretical curve by Equation (17) passes through the peak point of the axial stress–strain curve, the coordinate of the peak point was substituted into Equation (17):

(18) $$\sigma_{p}=E\left(\varepsilon_{p}-\varepsilon_{0}\right)\left\{1-\frac{1}{1+\exp\left[\lambda -\upsilon \left(\varepsilon_{p}-\varepsilon_{0}\right)\right]}\right\}$$

Meanwhile, the derivative of Equation (17) at the peak point is zero due to the geometrical characteristic of the theoretical curve; therefore,

(19) $$E-\left\{\frac{E}{1+\exp\left[\lambda -\upsilon \left(\varepsilon_{p}-\varepsilon_{0}\right)\right]}+\frac{\upsilon E\left(\varepsilon_{p}-\varepsilon_{0}\right)\exp\left[\lambda -\upsilon \left(\varepsilon_{p}-\varepsilon_{0}\right)\right]}{{\left[1+\exp\left(\lambda -\upsilon \left(\varepsilon_{p}-\varepsilon_{0}\right)\right)\right]}^{2}}\right\}=0$$

By solving the simultaneous equations of Equations (18) and (19), the mathematical expressions of parameters *υ* and *λ* can be determined.

(20) $$\upsilon =\frac{E}{E\left(\varepsilon_{p}-\varepsilon_{0}\right)-\sigma_{p}}$$

(21) $$\lambda =\ln\left[\frac{E\left(\varepsilon_{p}-\varepsilon_{0}\right)}{E\left(\varepsilon_{p}-\varepsilon_{0}\right)-\sigma_{p}}-1\right]+\upsilon \left(\varepsilon_{p}-\varepsilon_{0}\right)$$

### 3.3. Characterization of Residual Stage in Damage Constitutive Model

Equation (16) is just the damage evolution equation of the rock under the uniaxial condition based on the theory of fracture growth and the strain equivalence. This model considers that damage is the micro-deficiencies inside the rock material, and they cannot withstand any stress once formed, which means *σ*_1_ = 0 when *D* = 1. This obviously conflicts with the fact that the actual rock has residual strength. In this case, Shen [58] and Cao et al. [59] proposed that the damaged component of rock provided the residual stress *σ_r_*, as presented in Equation (22). In fact, there exist a great number of ways to consider the residual strength in the model such as using various correction factors with different definitions [34,35], while the core is to satisfy the relation that axial stress is equal to the residual strength when *D* is 1. By contrast, Equation (22) is more intuitive. That is, when the rock is completely damaged, the bearing capacity of the rock is provided by residual strength.

(22) $$\sigma =\left(1-D\right)E\varepsilon +D\sigma_{r}$$

Thus, Equation (17) can be recast as

(23) $$\sigma_{1}=E\left(\varepsilon_{1}-\varepsilon_{0}\right)-\frac{E\left(\varepsilon_{1}-\varepsilon_{0}\right)-\sigma_{r}}{1+\exp\left[\lambda -\upsilon \left(\varepsilon_{1}-\varepsilon_{0}\right)\right]}$$

Similarly, the mathematical expressions of parameters *υ* and *λ* can be recast. While it is interesting to note that the participation of rock residual strength shows no influence on the numerical determination of parameter *υ*, the mathematical expression of parameter *λ* has changed, which is presented in Equation (24).

(24) $$\lambda =\ln\left(\frac{E\left(\varepsilon_{p}-\varepsilon_{0}\right)-\sigma_{r}}{E\left(\varepsilon_{p}-\varepsilon_{0}\right)-\sigma_{p}}-1\right)+\upsilon \left(\varepsilon_{p}-\varepsilon_{0}\right)$$

After combining Equations (2) and (23), the whole constitutive model of rock can be expressed by the piecewise model as follows:

(25) $$\{\begin{array}{cc} \sigma_{1}=a\varepsilon_{1}^{b+1}+c\varepsilon_{1} & \varepsilon_{1}\leq \varepsilon_{cc}\\ \sigma_{1}=E\left(\varepsilon_{1}-\varepsilon_{0}\right)-\frac{E\left(\varepsilon_{1}-\varepsilon_{0}\right)-\sigma_{r}}{1+\exp\left[\lambda -\upsilon \left(\varepsilon_{1}-\varepsilon_{0}\right)\right]} & \varepsilon_{1}\geq \varepsilon_{cc} \end{array}$$

## 4. Model Verifications

Although the theoretical model of the rock constitutive relation was obtained, as presented in Equation (25), the correctness and accuracy are still not known, which need to be validated with experimental data. In this paper, both the uniaxial compression test results and triaxial compression test results were selected for model verification. In addition, the coefficient of determination *R*^2^ was used to assess the matching effect of the proposed model against the experimental data, which can be calculated by Equation (26).

(26) $$R^{2}=1-\frac{m-1}{m-2}\times \frac{\sum_{i=1}^{m}{\left(y_{{}_{test}}^{i}-y_{{}_{cal}}^{i}\right)}^{2}}{\sum_{i=1}^{m}{\left(y_{{}_{test}}^{i}-y_{ave}\right)}^{2}}$$

where *m* is the number of measured points in the axial stress–strain curve; *y_test_* and *y_cal_* are, respectively, the measured axial stress and the theoretical axial stress; *y_ave_* is the average of the *y_test_*.

### 4.1. Uniaxial Compression Test Verification

The uniaxial compression test results of five rock materials were used for validation. They were Sandstone [52], Beishan Granite [60], 130 m LdB Granite [49], Hwangdeung Granite, and Yeosan Marble [53]. From Equation (25), there are eight parameters that need to be determined. Taking the axial stress–strain curve of Sandstone for example, the specific procedure is shown as follows:

The first step is to locate the point of fracture closure stress in the axial stress–strain curve, i.e., distinguish model boundaries of Equation (25), and adopt the corresponding model expression. The values of fracture closure stress *σ_cc_* and fracture closure strain *ε_cc_* can be determined by the following methods: the fracture volume strain method [25], the axial strain curve method [61], the axial stiffness method [49], the axial strain response method [46], and the rock constitutive model method [62]. Any one of them, or other effective means, can be chosen according to the experimental conditions. Here, the fracture closure stress and the fracture closure strain have already been presented in the measured mechanical properties in the literature [52], and therefore, *ε_cc_* = 2.39 ×10^−3^ and *σ_cc_* = 12.6 MPa were directly adopted.

Then, the values of the tangent elasticity modulus *E* and the initial elasticity modulus *E_ini_* were determined from the axial stress–strain curve. In addition, *E* = 10.79 GPa and *E_ini_* = 1.99 GPa are given in the literature. By substituting *ε_cc_*, *σ_cc_*, *E,* and *E_ini_* into Equations (4), (7), and (8), the values of model parameters *a*, *b,* and *c* can be solved, wherein *a* = 0.759, *b* = 1.681, and *c* = 1.990. It should be noted that there exist certain differences between the calculated values and the fitted values in Figure 5 where *a_fitted_* = 0.957, *b_fitted_* = 1.479, and *c_fitted_* = 1.843, which are within the margin of error. Hence, the model of sandstone in the compaction stage is achieved, which is shown as:

(27) $$\sigma_{1}=0.759\times \varepsilon_{1}{}^{2.681}+1.99\varepsilon_{1}$$

Subsequently, the peak stress and strain in the axial stress–strain curve (*ε_p_* = 3.970 × 10^−3^ and *σ_p_* = 27.510 MPa) were gained for sandstone. The new origin *ε*_0_ can be fixed by Equation (12) when the values of *ε_cc_*, *σ_cc_*, and *E* are known, and the calculated *ε*_0_ = 1.220 × 10^−3^. According to Equations (20) and (21) and the coordinates of the peak point, the values of damage model parameters *υ* and *λ* can be gained, wherein *υ* = 5.046 and *λ* = 16.421. Therefore, the damage constitutive model of sandstone after the compaction stage is presented by Equation (28).

(28) $$\sigma_{1}=10790\times \left(\varepsilon_{1}-1.22\times 10^{-3}\right)\left\{1-\frac{1}{1+\exp\left[16.421-5.046\times \left(\varepsilon_{1}-1.22\times 10^{-3}\right)\right]}\right\}$$

**Figure 8:**
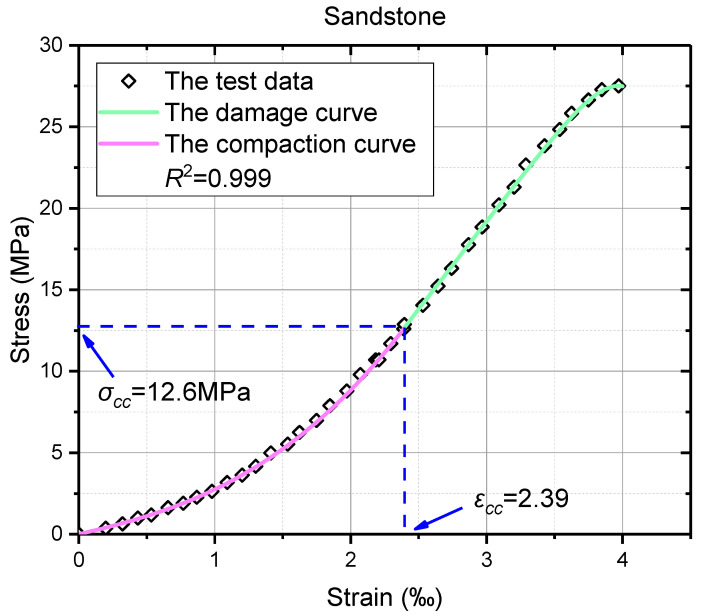
The comparison between the uniaxial test data and the model curve of sandstone.

The axial stress–strain curve of sandstone in pre-peak stage is depicted in Figure 8, in which the compaction curve is marked in light magenta while the damage curve is marked in prasinous. Fortunately, a strong consistency between the curve and test results is shown.

Finally, the coefficient of determination *R*^2^ is calculated. By substituting the values of the tested axial strains into Equations (27) and (28) successively, the calculated axial stresses can be obtained. The result is *R*^2^ = 0.999, verifying the validity of the proposed models. In addition, the fracture closure point where the damage curve and the compaction curve inosculate is smooth and continuous.

**Figure 9:**
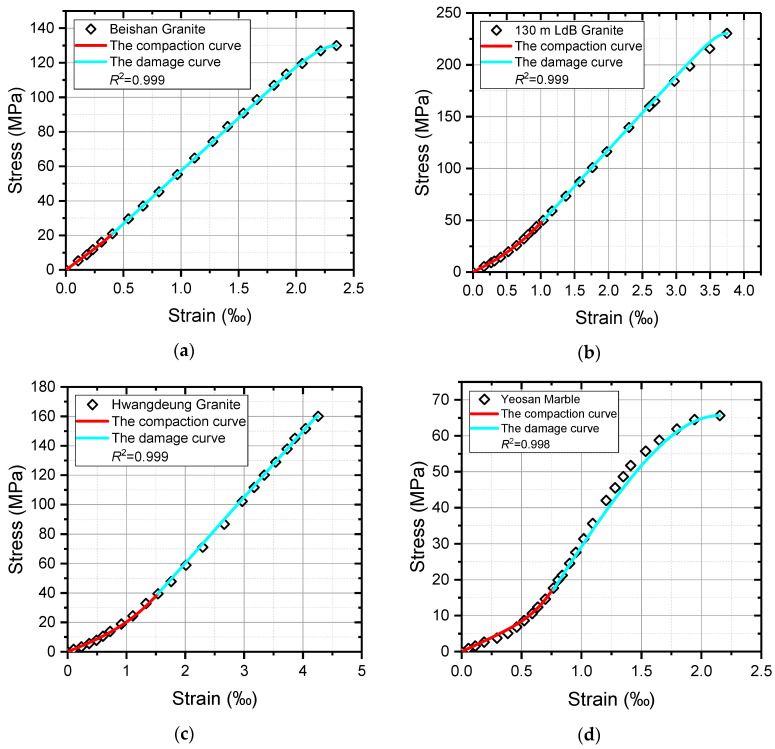
The comparison between uniaxial test data and model curves of various rocks. (**a**) Beishan Granite; (**b**) 130 m LdB Granite; (**c**) Hwangdeung Granite; (**d**) Yeosan Marble.

To avoid contingency and subjectivity of the comparison result in Figure 8, and explore the applicability of the proposed models to other rock materials, the theoretical curves of the other four rock materials were obtained according to the above procedure, which are plotted with test data in Figure 9. In addition, the necessary model parameters are presented in Table 1, wherein the red curves are the constitutive relations of rock materials in the compaction stage, while the cyan curves are the damage constitutive relations of rock materials after the compaction stage. Similarly, the values of *R*^2^ convincingly indicate significant agreements of the theoretical curves and test data.

### 4.2. Triaxial Compression Test Verification

**Figure 10:**
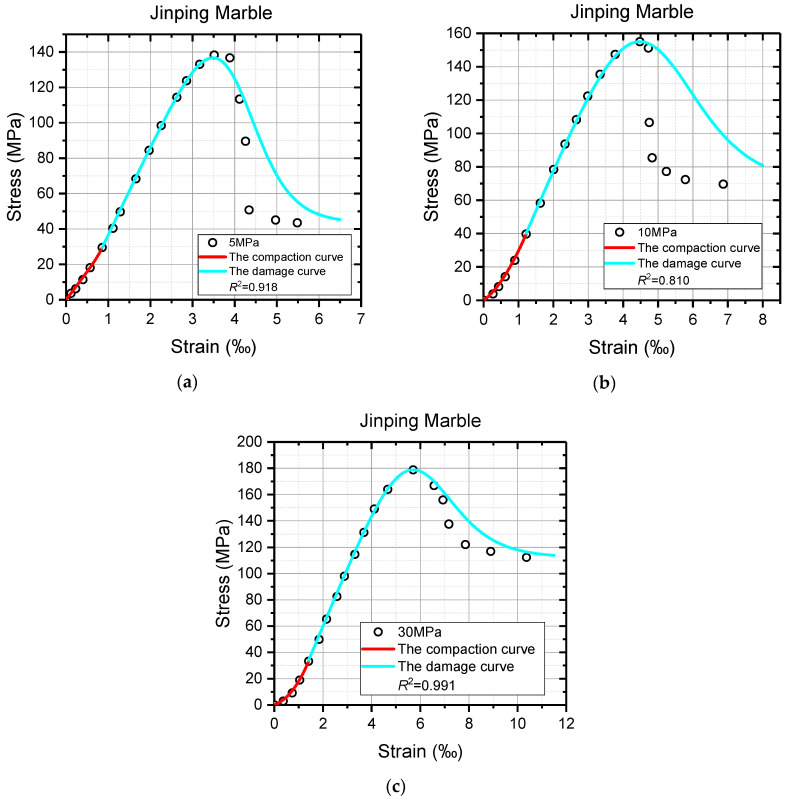
The comparison between partial triaxial test data and model curves of Jinping Marble. (**a**) Confining stress = 5 MPa; (**b**) confining stress = 10 MPa; (**c**) confining stress = 30 MPa.

**Figure 11:**
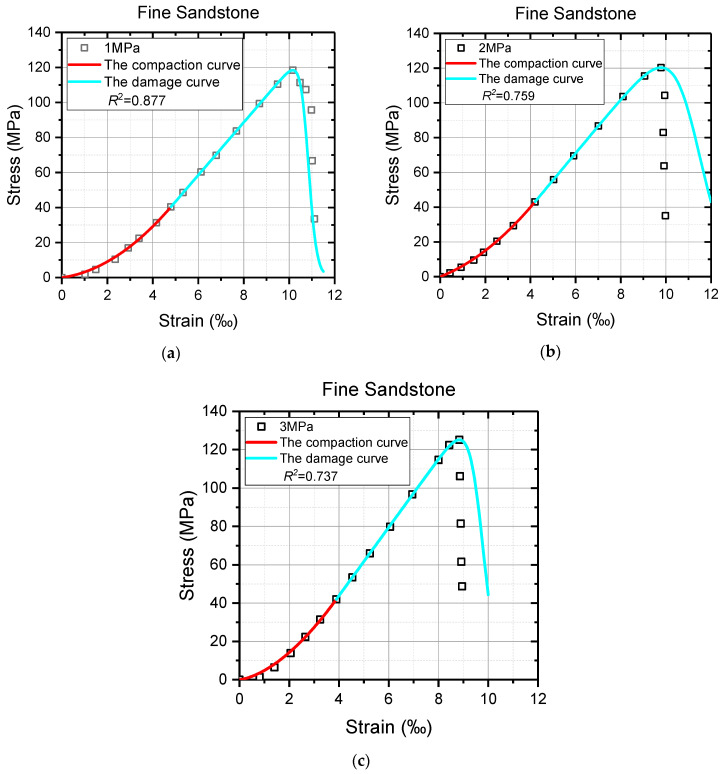
The comparison between partial triaxial test data and model curves of fine sandstone. (**a**) Confining stress = 1 MPa; (**b**) confining stress = 2 MPa; (**c**) confining stress = 3 MPa.

**Figure 12:**
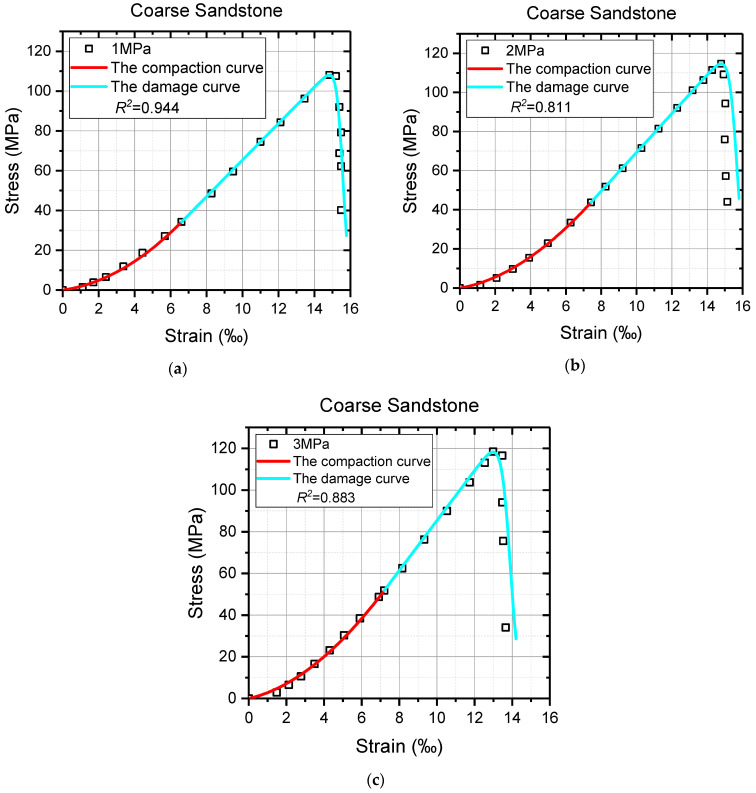
The comparison between partial triaxial test data and model curves of coarse sandstone. (**a**) Confining stress = 1 MPa; (**b**) confining stress = 2 MPa; (**c**) confining stress = 3 MPa.

Despite the models showing considerable efficacy in describing the constitutive relation of rocks under uniaxial compression, the feasibility of the proposed models in triaxial compression tests is still up for debate. Thus, the triaxial test results with obvious deformation characteristics of Jinping Marble under high confinements [63], and fine sandstone and coarse sandstone under low confinements [64] were utilized for further validation. The same parameter solution processes were carried out except for solving the values of *λ*, which were calculated by Equation (23) here, and the specific operations are not repeated. The model curves of marble and sandstone and the triaxial test data, as well as the values of the coefficient of determination *R*^2^, are shown in Figure 10, Figure 11 and Figure 12. In addition, the necessary model parameters are presented in Table 2. It must be noted that the residual strength in reference [64] is ambiguous due to the high strength and low confining pressure.

It can be seen from Figure 10, Figure 11 and Figure 12 that all the model curves generally agree well with the triaxial test data of sandstone and marble, especially the pre-peak stage of the axial stress–strain curves, which further illustrates the correctness and applicability of the model proposed in this study. From the view, the nonlinearity of the axial stress–strain curve in the compaction stage is delineated perfectly by the red compaction curves, and the deformation behaviors from the fracture closure to the peak are also successfully reflected by the cyan damage curves. The values of *R*^2^ are even up to 0.999 for these model curves and test data before the peak. By contrast, the agreements between the triaxial test data and the model curves after the peak are not so satisfactory, as shown in Figure 10b and Figure 11b,c. These theoretical curves significantly deviate from the test data in the post-peak stage, which causes the decrease in the value of *R*^2^. It is suspected that the practice of treating model parameter *ν* as a constant may be responsible for such a weak consistency between the model curve and test data after the peak. Although the proposed models cannot always agree well with the post-peak deformations of rock materials, they are still of great research significance and application value to the pre-peak characteristics of rock materials.

## 5. Conclusions

By considering the nonlinearity during the fracture closure and the fracture growth feature of rock failure, a new piecewise constitutive model was proposed to describe the whole axial stress–strain relation of rock materials in this study. The conclusions are as follows:

(1) According to the increasing trend of the fracture closure stage of the axial stress–strain curve, the nonlinearity characteristic during fracture closure was fitted by the power function, which was then used to deduce the compaction empirical model. The model parameters were solved by the initial modulus of elasticity and fracture closure stress and strain.
(2) The rock damage evolution was quantified by the fracture growth, and the damage constitutive model was derived based on the strain equivalence hypothesis to manifest the rock deformation after the fracture closure, which avoids the selection of the strength criterion for rock micro-units in damage statistical constitutive models. The model parameters were calculated by the derivative and the coordinate at the peak.
(3) The compaction empirical model and the damage constitutive model consist of the piecewise constitutive model representing the whole axial stress–strain relation of rock materials. Through the comparisons between the test data of uniaxial tests and triaxial tests and the model curves, the model validity was demonstrated. The model curves perfectly agree with the test data before the peak. In addition, the models are continuous and smooth at the curve intersection.

## Figures and Tables

**Table 1:** Model parameters for uniaxial tests.

Test Rocks	*a*	*b*	*c*	*ε_cc_*/10^−3^	*ε*_0_/10^−3^	*λ*	*υ*
Beishan Granite	75.579	3.729	49.045	0.404	0.064	16.416	6.059
130 m LdB Granite	14.739	1.463	32.652	1.036	0.332	23.527	6.019
Hwangdeung Granite	3.865	2.051	16.399	1.535	0.654	116.688	31.069
Yeosan Marble	21.860	3.996	15.582	0.765	0.442	4.216	1.962

**Table 2:** Model parameters for triaxial tests.

Test Rocks	*a*	*b*	*c*	*ε_cc_*/10^−3^	*ε*_0_/10^−3^	*λ*	*υ*	*σ_r_*/MPa
Jinping Marble 5 MPa	6.490	5.642	31.714	0.857	0.265	8.153	2.106	43.495
Jinping Marble 10 MPa	15.585	0.849	14.021	1.222	0.409	5.209	1.115	69.607
Jinping Marble 30 MPa	10.184	1.270	7.784	1.411	0.655	5.573	1.018	112.321
Fine sandstone 1 MPa	1.352	1.011	1.817	4.794	2.119	48.527	5.566	-
Fine sandstone 2 MPa	1.129	1.046	5.198	4.198	1.430	15.920	1.604	-
Fine sandstone 3 MPa	3.169	0.777	1.668	3.892	1.542	28.800	3.509	-
Coarse sandstone 1 MPa	0.539	1.039	1.342	6.608	2.875	72.564	5.716	-
Coarse sandstone 2 MPa	0.755	0.892	1.384	7.423	3.011	56.586	4.469	-
Coarse sandstone 3 MPa	0.890	0.904	1.886	7.200	2.882	45.500	4.137	-

## Data Availability

Some or all data, models, or code that support the findings of this study are available from the corresponding author upon reasonable request.
